# Next-generation transcriptome sequencing of the premenopausal breast epithelium using specimens from a normal human breast tissue bank

**DOI:** 10.1186/bcr3627

**Published:** 2014-03-17

**Authors:** Ivanesa Pardo, Heather A Lillemoe, Rachel J Blosser, MiRan Choi, Candice A M Sauder, Diane K Doxey, Theresa Mathieson, Bradley A Hancock, Dadrie Baptiste, Rutuja Atale, Matthew Hickenbotham, Jin Zhu, Jarret Glasscock, Anna Maria V Storniolo, Faye Zheng, RW Doerge, Yunlong Liu, Sunil Badve, Milan Radovich, Susan E Clare

**Affiliations:** 1 Department of Surgery, Feinberg School of Medicine, Northwestern University, 303 East Superior Street, Chicago, IL 60611, USA; 2 Department of Surgery, Indiana University School of Medicine, 980 West Walnut Street, Indianapolis, IN 46202, USA; 3 Susan G. Komen for the Cure Tissue Bank at the IU Simon Cancer Center, 550 University Boulevard, Indianapolis, IN 46202, USA; 4 Department of Medicine, Indiana University School of Medicine, 535 Barnhill Drive, Indianapolis, IN 46202, USA; 5 Cofactor Genomics, LLC, 3139 Olive Street, St. Louis, MO 631036, USA; 6 Department of Statistics, Purdue University, 150 North University Street, West Lafayette, IN 47907, USA; 7 Department of Medical and Molecular Genetics, Indiana University School of Medicine, 410 West 10th Street, Indianapolis, IN 46202-5122, USA; 8 Department of Pathology, Indiana University School of Medicine, 350 West 11th Street, Indianapolis, IN 46202-4108, USA

## Abstract

**Introduction:**

Our efforts to prevent and treat breast cancer are significantly impeded by a lack of knowledge of the biology and developmental genetics of the normal mammary gland. In order to provide the specimens that will facilitate such an understanding, The Susan G. Komen for the Cure Tissue Bank at the IU Simon Cancer Center (KTB) was established. The KTB is, to our knowledge, the only biorepository in the world prospectively established to collect normal, healthy breast tissue from volunteer donors. As a first initiative toward a molecular understanding of the biology and developmental genetics of the normal mammary gland, the effect of the menstrual cycle and hormonal contraceptives on DNA expression in the normal breast epithelium was examined.

**Methods:**

Using normal breast tissue from 20 premenopausal donors to KTB, the changes in the mRNA of the normal breast epithelium as a function of phase of the menstrual cycle and hormonal contraception were assayed using next-generation whole transcriptome sequencing (RNA-Seq).

**Results:**

In total, 255 genes representing 1.4% of all genes were deemed to have statistically significant differential expression between the two phases of the menstrual cycle. The overwhelming majority (221; 87%) of the genes have higher expression during the luteal phase. These data provide important insights into the processes occurring during each phase of the menstrual cycle. There was only a single gene significantly differentially expressed when comparing the epithelium of women using hormonal contraception to those in the luteal phase.

**Conclusions:**

We have taken advantage of a unique research resource, the KTB, to complete the first-ever next-generation transcriptome sequencing of the epithelial compartment of 20 normal human breast specimens. This work has produced a comprehensive catalog of the differences in the expression of protein-coding genes as a function of the phase of the menstrual cycle. These data constitute the beginning of a reference data set of the normal mammary gland, which can be consulted for comparison with data developed from malignant specimens, or to mine the effects of the hormonal flux that occurs during the menstrual cycle.

## Introduction

In 1997, the National Cancer Institute (NCI) convened a meeting of researchers from academia, industry and government, and representatives of the patient advocate community. The purpose of the meeting was to identify deficiencies that would have to be addressed if we are to continue and accelerate progress in treating breast cancer, and ultimately, to prevent this disease. Thirteen deficiencies were identified, the first of which was as follows:

‘*Our limited understanding of the biology and developmental genetics of the normal mammary gland is a barrier to progress. …it is now clear that a more complete understanding of the normal mammary gland at each stage of development—from infancy through adulthood—will be a critical underpinning of continued advances in detecting, preventing, and treating breast cancer*[1]*’.*

The ideal approach to end the scourge of breast cancer would be to prevent it. Annual age-adjusted breast cancer incidence rates in the United States are a testament to the lack of effective prevention strategies [2]. Current breast cancer prevention strategies fall into one of three categories: lifestyle modification, surgical intervention, and chemoprevention. Lifestyle modifications are directed at women with the general population risk of breast cancer, and modifications include limiting postmenopausal weight gain and moderation of alcohol intake. Surgical intervention, by convention, has been limited to those women estimated to be at substantially increased risk of breast cancer, including women with known or suspected germline mutations in *BRCA1* or *BRCA2*, or a family history of breast and/or ovarian cancer among first- and second-degree relatives. Surgical interventions include bilateral prophylactic mastectomy, bilateral salpingo-oophorectomy or a combination of both procedures. Multiple chemoprevention trials examining the efficacy of tamoxifen (for example, NSABP-P1, IBIS-1), raloxifene (MORE, CORE, STAR) and aromatase inhibitors have been completed [3-9]. All chemopreventative agents identified to date, with a single exception, were introduced into the clinic as breast cancer treatments; a significant reduction in the rate of contralateral breast cancer in treated patients was used as an indication that these treatments also act to prevent breast cancer. Few, if any, interventions are based on an understanding of breast cancer risk or of how risk is transduced at the molecular level.

The Susan G. Komen for the Cure Tissue Bank at the Indiana University (IU) Simon Cancer Center (KTB, The Bank) was established expressly as a resource to be used to address the deficiency identified by the NCI’s Progress Review Group and stated above [10]. To the best of our knowledge, the KTB has the largest and most varied collection of normal breast tissue in the world. The KTB was organized as a clinical trial, and specimens are obtained under broad consent. Healthy volunteer women are recruited by flyer, workplace newsletter, and email solicitation by friends and acquaintances. Donors present to a clinic on a weekend day. They fill out a questionnaire, which provides detailed information on their menstrual history, reproductive history, personal health history, medication usage and family history of breast, ovarian and other cancers. Blood is obtained and processed for leukocyte DNA, as well as for serum and plasma. Breast tissue acquisition is done utilizing a 10-gauge breast biopsy system. Three tissue cores are fresh frozen in liquid nitrogen within five minutes of extraction and a fourth is fixed in formalin and paraffin-embedded (FFPE).

As a first initiative toward a molecular understanding of the biology and developmental genetics of the normal mammary gland, the effect of the menstrual cycle and hormonal contraception on DNA expression in the normal breast epithelium was examined. The normal epithelium was chosen for sequencing for two major purposes: 1) to determine how DNA expression changes as a consequence of the menstrual cycle in the functional unit of the breast, that is, the ductal/lobular epithelium; and 2) anticipating that this sequencing information would be used as a normal control in breast cancer experiments, the epithelium was deemed to be the best comparator as it is hypothesized that breast cancer originates in the terminal ductal lobular unit of the epithelium. This manuscript reports the findings for protein-coding genes.

The human mammary gland undergoes rounds of proliferation, differentiation, and regression/involution in response to cyclic fluctuations in the concentration of ovarian steroidal hormones. Much of what is known about the specific changes in the normal mammary gland as a function of these hormones comes from the study of other mammals [11,12]. A large proportion of our knowledge of menstrual cycle effects in the human breast comes from histologic observations [13], and studies of markers of proliferation and apoptosis. It is known that the mitotic index is low during the follicular phase with the peak of mitotic activity occurring in the mid to late luteal phase [14,15]. In the event that a pregnancy does not occur, to prevent hyperplasia following the cell proliferation of the luteal phase, apoptosis must be activated to clear the superfluous cells. There is evidence to suggest that apoptosis occurs during the luteal phase [16], while other researchers have found no differences between the phases [17]. With the identification of the breast stem cell, attention has turned to the effect of the menstrual cycle on this cell and its niche. Asselin-Labat and colleagues demonstrated markedly decreased murine mammary stem cell numbers and outgrowth potential as a consequence of the elimination of steroidal hormones following ovariectomy [18]. Joshi *et al.* observed that the number of mouse ‘mammary stem cell-enriched basal cells’ increases at diestrus or following the administration of exogenous progesterone [19]. Breast malignancies also appear to be affected. Murine breast cancers fluctuate in size as a function of the menstrual cycle, thought likely a consequence of the periodic proliferation and apoptosis driven by the hormone flux [20]. Human tumors show increased proliferative activity as a function of the menstrual cycle [21], but measures of apoptosis remain unchanged [22].

## Materials and methods

All studies were approved by the Indiana University Institutional Review Board (IRB-04, protocol number 0709–17; IRB-01, protocol number 1110007030). All research was carried out in compliance with the Helsinki Declaration. Donors provide broad consent for the use of their specimens in research. The consent document informs the donor that the donated specimens and medical data will be used ‘for the general purpose of helping to determine how breast cancer develops’. It is explained in the consent that the exact laboratory experiments are unknown at the time of donation, and that proposals for use of the specimens will be reviewed and approved by a panel of ‘independent researchers’ before specimens and/or data are released for research purposes.

Premenopausal donors to the KTB were identified by a query of the Bank’s database. Hematoxylin and eosin-stained sections of the FFPE tissue of the identified donors were reviewed and tissue was graded on the basis of the abundance of epithelium within the section. Only cores containing abundant epithelium were considered for this study. Based on dates, the specimens of nine women in the follicular phase of the menstrual cycle and five in the luteal phase were chosen (Table 1). Six donors using hormonal contraception at the time of donation were also included (Table 1). Whole blood obtained from 19 of the 20 donors at the time of tissue donation was processed for serum. Estradiol, estriol, luteinizing hormone (LH) and progesterone concentrations were determined by the IU Health Pathology Laboratory using a Beckman Unicel DxI 800 Immunoassay System (Beckman Coulter, Brea, CA, USA). The phase of the menstrual cycle was verified by serum progesterone concentration (Table 1). The epithelium of these 20 specimens was microdissected from multiple 8 micron thick frozen tissue sections. Total RNA extracted from the tissue was subsequently depleted of rRNA via locked nucleic acid probes (see Additional file 1). This enabled profiling of both poly-A and non-poly-A RNA species. Barcoded cDNA libraries from the 20 normal breast epithelia were prepared and sequenced on an Applied Biosystems (AB) SOLiD 3 or SOLiD 4 platform (Life Technologies, Foster City, CA, USA) (Table S1 in Additional file 2). Whole transcriptome sequencing (RNA-Seq) reads for each sample were then mapped to the human genome (hg19) using the LifeScope software version 2.5.1 (Life Technologies) and Binary Alignment/Map (BAM) files were generated. The files can be accessed using the Database of Genotypes and Phenotypes (dbGaP) [23,24], study accession number phs000644.v1.p1. Read counts for each gene were derived from the output BAM files using the RefSeq database (UCSC Genome Brower) as the gene model. The total number of reads and the mapped reads are provided in Table S2 in Additional file 2; the raw read counts of the individual genes are listed in Table S3 in Additional file 2.

**Table 1:** Age, menstrual phase data and hormonal contraception (HC) formulation

**Sequencing ID**	**Age**	**Menstrual day**	**HC**	**HC type**	**F or L**	**Estradiol pg/mL**	**Progesterone ng/mL**	**LH milliunits/mL**
Normal_1	37	5	no		F	49	0.5	5.7
Normal_2	20	14	yes	Tri-Legest Fe	-	32	1.5	9.6
Normal_3	30	13	no		F	32	<0.01	3.6
Normal_4	44	19	yes	Ortho Tri-Cyclen Lo	-	432	0.4	5.1
Normal_5	40	9	no		F	28	0.5	5.6
Normal_6	27	27	yes	Nuvaring	-	N/A	N/A	N/A
Normal_7	23	29	no		L	71	6.1	3.2
Normal_8	27	25	no		L	82	3.7	4.9
Normal_9	39	4	no		F	198	0.1	N/A
Normal_10	38	26	no		L	131	21.4	3
Normal_11	22	28	yes	Necon1-35	-	36	1.1	0.2
Normal_12	45	27	no		L	47	2.9	3.6
Normal_13	19	7	yes	Zenchent	-	26	0.7	2.4
Normal_14	36	2	no		F	53	0.6	4.4
Normal_15	26	9	no		F	43	0.9	10.3
Normal_16	22	29	no		L	136	12.4	5.3
Normal_17	46	6	no		F	121	0.5	5.6
Normal_18	31	29	yes	LoEstrin 24	-	34	1.1	2
Normal_19	29	2	no		F	61	0.6	7.9
Normal_20	21	7	no		F	43	0.3	3.5
**HC type**								
Tri-Legest Fe	1 mg norethindrone acetate and 20 mcg ethinyl estradiol × 1 week; 1 mg norethindrone acetate and 30 mcg ethinyl estradiol × 1 week; 1 mg norethindrone acetate and 35 mcg ethinyl estradiol × 1 week							
Ortho Tri-Cyclen Lo	0.180 mg of norgestimate and 0.025 mg ethinyl estradiol × 1 week; 0.215 mg of norgestimate and 0.025 mg ethinyl estradiol × 1 week; 0.250 mg of norgestimate and 0.025 mg of ethinyl estradiol × 1 week							
Nuvaring								
Necon1-35	Norethindrone 1 mg, ethinyl estradiol 35 mcg							
Zenchent	0.4 mg norethindrone and 0.035 mg ethinyl estradiol							
LoEstrin 24	1 mg norethindrone acetate and 20 mcg ethinyl estradiol							

Menstrual day was calculated from information provided by the donor on her questionnaire and verified by serum progesterone concentration. F, follicular; L, luteal; LH, luteinizing hormone.

The original data comprised 25,203 sequenced genes, many of which exhibit very low expression levels. Omitting low-expression genes that contribute little to the analysis yields a more powerful statistical test overall, that is, the asymptotic theory required by the statistical tests is satisfied. Genes that had average counts greater than 5 across all samples were retained for analysis. A total of 7,208 genes were removed based upon this criterion (28.6% of the original number); 17,995 genes remained for analysis.

### Statistical analysis

Differential expression (DE) was tested using the Bioconductor package *edgeR* in R (v. 2.15). A negative binomial (NB) distribution was employed to model the count data generated from the RNA-Seq experiments.

Three similar general linear models were employed to test a set of three hypotheses. In addition to modeling the effects of membership in the groups of interest (luteal phase, follicular phase, and contraceptives), terms are also included to model the effects of batch membership. Specifically, Batch 1 (samples 1 through 10) acts as the baseline for comparison, and the coefficients for Batch 2 (samples 11 through 20 except 19) and Batch 3 (sample 19) indicate departures from that baseline. These terms ensure that the systematic differences in expression that are present between batches, including differences due to single-end versus paired-end reads, are not falsely attributed to differential expression between the actual groups of interest. Details are provided in Additional file 1.

There are situations when a set of genes is highly expressed in one sample but not in another. When this occurs, the remainder of the genes in the first sample would be ‘under-sampled’, and thus creates a potential bias due to its particular RNA composition. To prevent this occurring, and from skewing the DE analysis and results, the data was normalized using an empirical approach that estimates bias [23]. The scaling factors that were estimated ranged from 0.4402 to 1.3760 across the 20 samples; the departure of these factors from 1 indicates the presence of compositional differences between libraries.

The NB model includes ψ_*g*_ as a dispersion parameter. Initially a common dispersion was estimated, which is the average ψ_*g*_ across all genes, and then this was extended by estimating a separate dispersion for each individual gene. This was done using an empirical Bayes method that ‘squeezes’ the gene-wise dispersions toward the common dispersion, thus allowing for information borrowing from other genes [25].

### Adjustments for multiple testing

Since a separate statistical test is performed for all of the 17,995 genes, it is necessary to adjust the *P* values for multiple testing (to control the Type I error rates across the ‘family’ of genes rather than for ‘each’ gene). This was accomplished using the Benjamini-Hochberg procedure for controlling the expected proportion of incorrectly rejected null hypotheses, also known as the false discovery rate (FDR) [25].

Residual RNA from specimens 11 to 20 was utilized to validate the sequencing findings. TaqMan qPCR was performed for 29 genes (Additional file 1). qPCR reactions were run on an ABI 7900HT Real-Time PCR System and data analyzed using the SDS2.3 and DataAssist v2.0 software from Applied Biosystems.

### Functional analysis

Networks and functional analyses were generated through the use of Ingenuity Pathway Analysis (IPA) (Ingenuity systems, [26]) and the database for annotation, visualization and integrated discovery (DAVID) [27,28] bioinformatics resources.

### Ki-67 immunohistochemistry

Tissue cores were placed in 10% neutral buffered formalin within 5 minutes of acquisition and delivered to IU Health Pathology for routine paraffin embedding. Sections 3 to 5 microns thick were deparaffinized and hydrated to running water. Antigen retrieval was carried out in the pretreatment module (DAKO, Carpinteria, CA, USA) using low pH target retrieval (DAKO). All staining was performed on the AutoStainer Plus (DAKO). Sections were incubated with 3% H_2_O_2_ for 5 minutes and subsequently exposed to the primary antibody, Ki-67 (Mib-1, DAKO), for 20 minutes. Horseradish peroxidase-labeled secondary antibody (EnVision™/HRP) was placed on the tissue for 20 minutes followed by 3,3′-diaminobenzidine (DAB) + chromogen for 10 minutes. Sections were counterstained with EnVision™ Flex Hematoxylin (DAKO). The pathologist (SB) was blinded as to the phase of the menstrual cycle or to the use of hormonal contraception (HC). Each section was classified from grade 1 to 4 where 1 is the lowest number of cells stained/slide and 4 is highest number of cells stained/slide.

## Results

### Gene expression differences between the follicular and luteal phases of the menstrual cycle

Some 255 genes representing 1.4% of all genes were deemed to have statistically significant differential expression between the two phases of the menstrual cycle (Table S4 in Additional file 2). A total of 221 of the genes have higher expression during the luteal phase.

**Figure 1:**
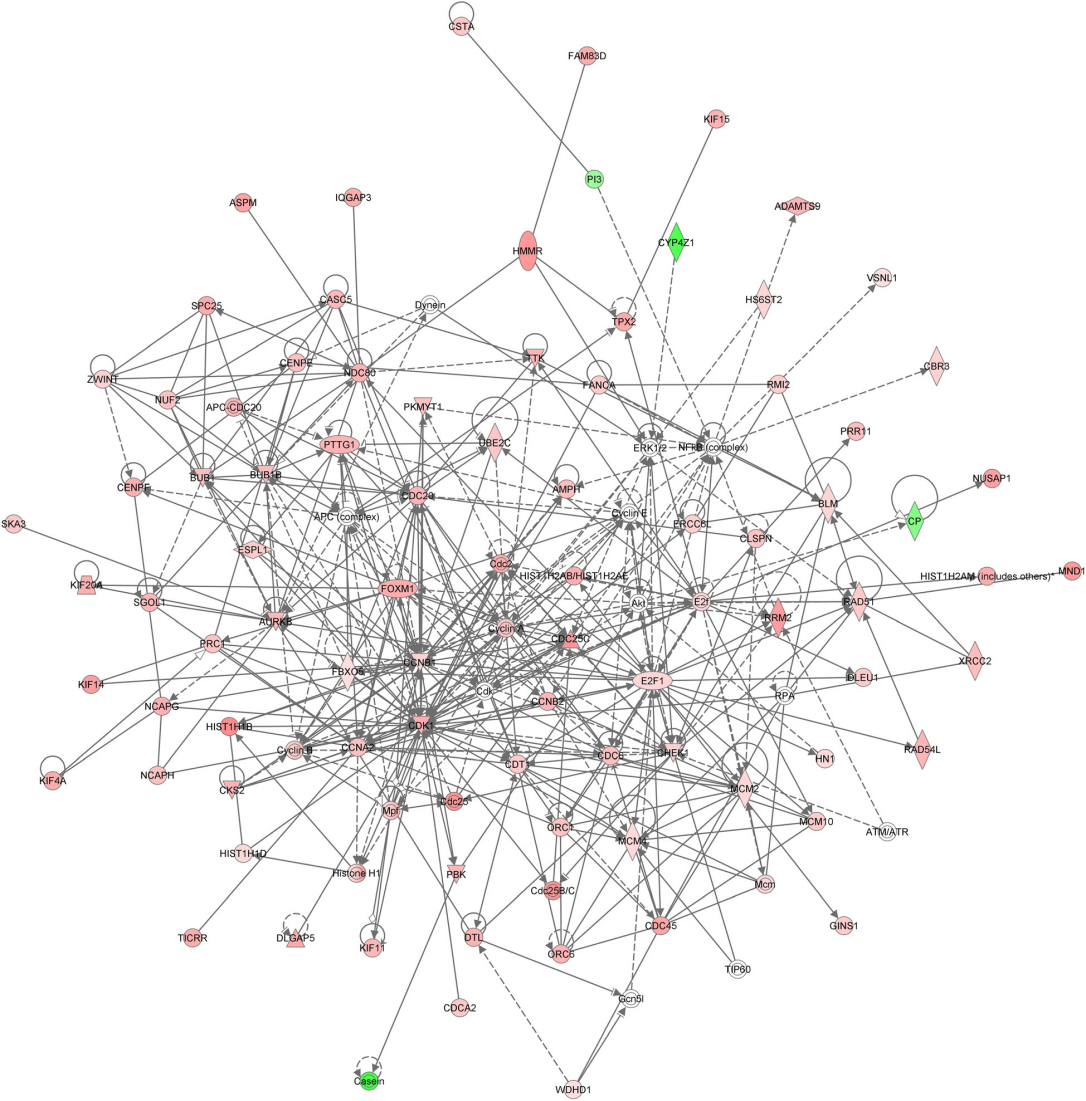
**Ingenuity Pathway Analysis networks.** The three most significant networks as determined by Ingenuity Pathway Analysis were merged. The top functions represented in this figure are cell cycle; cellular assembly and organization; DNA replication, recombination, and repair.

The genes with significant differential expression were analyzed using IPA. The molecular and cellular functions enriched in the gene list are provided in Table 2. These functions occur during the luteal phase. The top three networks as determined by IPA were merged and are presented in Figure 1. A total of 151 of the 255 genes are periodically expressed in the cell cycle as determined by comparison to lists generated in HeLa cells [29], foreskin fibroblasts [30], immortalized keratinocytes [31] and osteosarcoma [32] cell lines. Genes are displayed by the cell cycle phase in which they are expressed as well as by Gene Ontology (GO) biologic process terms in Table 3. GO biologic processes were ranked by statistical significance by DAVID; the top 30 are listed in Table S5 in Additional file 2.

**Table 2:** Biologic functions in the luteal phase

**Function**	***P *** **value**
Cell cycle	1.36E-28-1.22E-02
Cellular organization and assembly	1.36E-28-1.22E-02
DNA replication, recombination and repair	1.36E-28-1.22E-02

Top biologic functions of differentially expressed genes as determined by Ingenuity Pathway Analysis.

**Table 3:** Cell cycle phase and function

	**G1/S**	**S**	**G2**	**G2/M**	**M/G1**
DNA replication initiation	CDC45L				MCM10
	CDC6				
	MCM2				
	MCM4				
	ORC1				
	ORC6				
	CDT1				
Nucleotide metabolism				DTYMK	
				RRM2	
DNA replication and repair	BRIP1	TYMS	TOP2A	FANCA	
	BLM			RAD54L	
	DNA2			NEIL3	
	RAD51			PTTG1	
	EXO1			POLQ	
	POLE2			TRIP13	
	PCNA			KIAA0101	
	UNG				
	BRCA1				
	GINS2				
	DTL				
Chromatin assembly and disassembly	HELLS	HIST1H1B	HJURP	ASF1B	
	EZH2	HIST1H1D			
		HIST1H2AB			
		HIST1H2AH, HIST1H2AK, HIST1H2AM, HIST1H2AG,			
		HIST1H2AL			
		HIST1H2AJ			
		HIST1H2BH			
		HIST1H2BF, HIST1H2BE			
		HIST1H2BL			
		HIST1H2BM			
		HIST1H2BN			
		HIST1H2BO			
		HIST1H2AD, HIST1H3C, HIST1H3F, HIST1H3G, HIST1H3B, HIST1H3H, HIST1H3J, HIST1H3A, HIST1H3D			
		HIST1H4L, HIST1H4F, HIST1H4A			
Cell cycle regulation-interphase	CLSPN			CCNA2	
	E2F1			CKS2	
	CHEK1			MKI67	
Cell cycle regulation-mitosis	FBXO5			FOXM1	CCNB1
				CCNB2	
				CDC20	
				CDC25C	
				CIT	
				MELK	
				PKMYT1	
				UBE2C	
Microtubules and spindle formation				KIF11	KIF18A
				KIF14	KIF4A
				KIF15	
				KIF23	
				KIF2C	
				SPAG5	
				TPX2	
Spindle regulation				BUB1B	ASPM
				BUB1	
				AURKB	
				PLK4	
				PRC1	
				TTK	
Chromosome segregation	NDC80	ZWINT	NUF2	BIRC5	ESPL1
	SPC25		NCAPG	CENPE	CENPF
	DSCC1		NCAPH	DLGAP5	
			SGOL1	KIFC1	
			CASC5	NUSAP1	
Cytoskeleton	AMPH			CKAP2L	
Nuclear membrane					LAMB1
Transcription	ATAD2			MLF1IP	
	CDCA7				
	ZNF367				
Nuclear division			CDCA2	ANLN	
			FAM83D	CDC20	
				CDCA3	
GTPase activator activity				DEPDC1	
				DEPDC1B	
				ARHGAP-11A	
				IQGAP3	
Cytokinesis				PBK	RACGAP-1
				ECT2	
				KPNA2	
Membrane transport		ABCC2			
Metabolic process				HMMR	CBR3
				HSPH1	ELOVL6
				SRD5A1	

Differentially expressed genes were allocated to a phase of the cell cycle by referring to phase allocations for HeLa cells [29], foreskin fibroblasts [30], immortalized keratinocytes [31] and osteosarcoma cells [32]. Functions were assigned by referring to Bar-Joseph *et al.*[30], the database for annotation, visualization and integrated discovery (DAVID) and Ingenuity Pathway Analysis (IPA). Table modeled after Table S3 in Additional file 2 in reference [30].

A closer look at several of these genes and the processes they are involved in as well as non-cell cycle-controlled genes follows.

### Luteal phase genes

**Figure 2:**
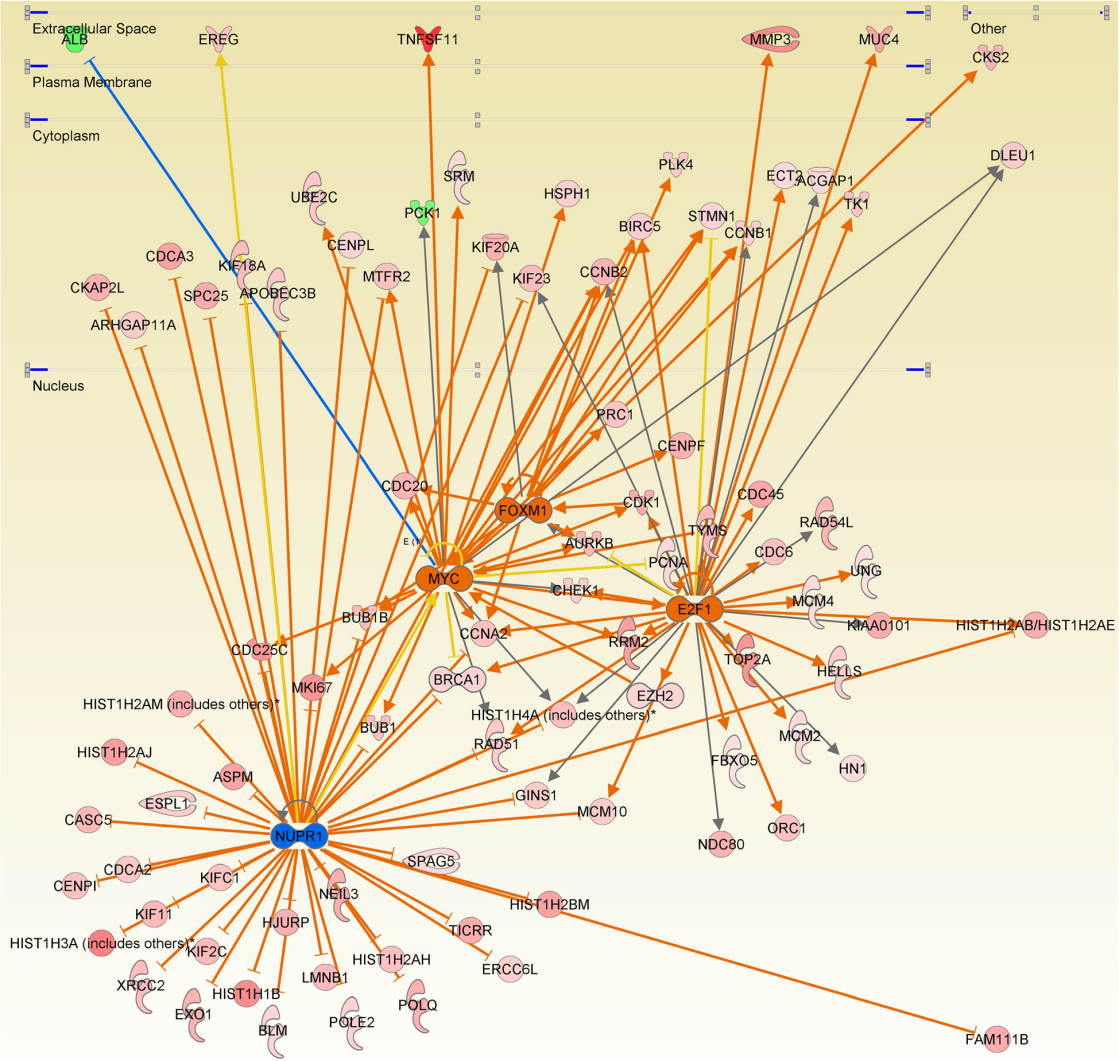
**Transcription factor targets.** Ingenuity Pathway Analysis upstream analysis was employed to determine the transcription factor (TF) pathways activated and inhibited during the luteal phase. This figure displays the E2F1, MYC, FOXM1 And NUPR1 targets whose expression changes during the luteal phase. Genes with increased expression in the luteal phase are indicted in red, those decreased in green. Orange TFs are activated and blue are inhibited. Line color indicates the following: orange: activation, blue: inhibition, yellow: findings inconsistent with the state of the downstream molecule, and grey: an effect not predicted.

Two of the major cellular events occurring during the cell cycle are DNA replication and mitosis. These events must occur at a specific time within the cycle and be successfully completed before progression to the next phase. Transcription of the genes that control or execute these functions is initiated by transcription factors. Both *E2F1* and *FOXM1* have higher expression during the luteal phase. Upstream analysis by IPA (Table S6 in Additional file 2) predicts E2F1 and MYC, and FOXM1 (Table S7 in Additional file 2) are activating gene transcription during the luteal phase while NUPR1 is the most significantly inhibited releasing its suppression of transcription (Figure 2).

### DNA replication

#### Initiation of DNA replication at the replication origins

There are four phases that occur during the initiation of DNA synthesis: recognition of the replication origins, assembly of the pre-replication complexes (pre-RC), activation of the DNA helicase(s) and loading of the replicative enzymes [33]. DNA becomes licensed for replication during late mitosis or the early G_1_ phase by the formation of pre-RC on replication origins [34]. Origins of replication are bound by origin replication complex (ORC)1-6 proteins and these form the platform for the loading of the minichromosome maintenance (MCM) proteins by CDC6 and CDT 1 to form the pre-RC. The expression of *ORC1*, *ORC6*, *CDC6*, *CDT1*, *MCM2*, and *MCM4* are increased during the luteal phase in the normal breast epithelium. The CMG complex consisting of the CDC45, MCM2-7 and the GINS proteins functions as a helicase. The expression of *CDC45*, *MCM2* and *4* and *GINS1* and *2* was observed to be increased during the luteal phase. With regard to the final step, the replicative enzymes, *PCNA* and *POLE* were increased in the luteal phase.

### DNA damage

BRCA1, RAD51, RAD54L, CHEK1, XRCC2, HJURP, RMI2 and BLM are involved in homologous recombination [35], and the expression of all seven genes increases during the luteal phase. BRIP1, which interacts with the BRCT repeats of BRCA1 and is important to BRCA1 function, is also increased [36]. *HMMR* and *MAD1L1* are both more highly expressed in the luteal phase. The proteins encoded by these genes associate in protein complexes with BRCA 1 and BRCA2 [37]. Base excision repair (BER) is represented by *NEIL3*[38], *POLE2, UNG* and *PCNA*. Fanconi anemia proteins are required for the repair of DNA cross-links [39]; *FANCA* expression is higher in the luteal phase. *EXO1*, the protein product of which is involved in DNA end resection and double-strand break repair, is increased during the luteal phase. DNA polymerase *POLQ* is more highly expressed during the luteal phase. This polymerase has the unique ability to bypass blocking lesions such as abasic sites and thymine glycols [38]. It is also able to extend unpaired termini [38].

### Mitosis

The terms associated with the gene group with the highest enrichment score as determined by the gene functional classification tool in DAVID are M phase and mitosis. Forty-seven (21%) of the 221 genes with increased levels of expression during the luteal phase are involved in mitosis. A list of the genes involved in mitosis and increased in expression during the luteal phase is provided in Table S8 in Additional file 2. While not intended to be exhaustive, a survey of some of the functions during mitosis of the encoded gene are also provided in the table. Recent studies employing small interfering RNA (siRNA) and proteomic strategies have enabled the kinetochore proteins to be assigned to complexes with specific functions at the kinetochore [33-35]. Using this information it was possible to assign three genes increased during the luteal phase to the Ndc80 complex, two to the chromosome passenger complex (CPC), three to the constitutive centromere-associated network (CCAN) and three to the spindle assembly checkpoint (SAC). Nine kinesin motor protein genes also demonstrate increased expression.

### Paracrine signaling

*RANKL*, *WNT4* and *EREG* are all overrepresented during the luteal phase.

### Hormones

*GHR* expression is increased during the luteal phase as are *PTHLH* and *SRD5A1*. SRD5A1 catalyzes the conversion of progesterone to the 5alpha-pregnanes, which are mitogens in the normal human breast [40]. It also converts testosterone to dihydroxytestosterone, which has distinctly anti-proliferative effects in the breast [41]. PTHLH promotes nipple formation and mammary duct branching during embryogenesis and has an important role in calcium transport in the lactating gland [42]. *ESRRB* encodes the estrogen-related receptor beta, an orphan nuclear receptor. This protein would seem to have disparate functions in that it is a key regulator of embryonic stem cell self-renewal in the mouse [43,44], however, its ortholog functions as a metabolic switch during development in the *Drosophila*[45].

### Matrix metalloproteinases

*MMP3* and *ADAMTS9* are both higher in expression during the luteal phase. MMP3 plays a role in mammary gland branching morphogenesis [46,47].

### Follicular phase genes

Thirty-four of the 251 differentially expressed genes show increased expression during the follicular phase. They can be assigned to categories based on function. Two are associated with fat droplets in milk: *BYN1A1*, *MUC15*. Three genes encode ion channels or transporters: *GLRA3*, *NHEDC1*, and *KCNT2*. Two genes are associated with a fully differentiated phenotype: *HOXB6*, *TFCP2L1*. A number of genes encode proteins with metabolic functions: *CYP4Z1*, *CYP4X1*, *HMGCS2*, and *PCK1*. PCK1 expression in the normal mammary gland results in gluconeogenesis and glycerolneogenesis [48]. PCK1*-*dependent glyceroneogenesis may contribute to the formation of milk triglycerides in epithelial cells during lactation [49]. PI3 is a serine protease inhibitor that has antibacterial activity against Gram-positive and Gram-negative bacteria as well as fungal pathogens on epithelial surfaces [50].

### Effect of hormonal contraception

*CCDC144A* was the only gene differentially expressed between the hormone contraceptive group and luteal phase group (Table S9 in Additional file 2). This gene encodes a protein of unknown function.

### qPCR validation

Twenty-nine genes were selected for qPCR validation; the expression of 27 of the 29 was validated (Table 4).

**Table 4:** Validation cohort

**Gene**	**logFC (RNA-Seq)**	***P *** **value**	**Fold change (qPCR biological validation)**	**Validation status**
*AURKB*	1.694476904	1.10E-05	8.56	V
*BRCA1*	1.042815222	4.95E-06	2.39	V
*BUB1*	1.526455024	2.82E-07	2.95	V
*BUB1B*	1.602146974	8.86E-07	3.87	V
*CCNB1*	1.055253675	9.55E-05	2.73	V
*CDC25C*	2.501848634	4.13E-11	6.1	V
*CDC6*	1.49654134	2.33E-08	3.76	V
*CDK1*	1.965582801	6.77E-12	6.33	V
*CENPE*	1.462857192	1.39E-06	3.05	V
*CLSPN*	1.383625655	6.57E-06	6.07	V
*E2F1*	0.996887203	0.000372	3.06	V
*ECT2*	0.872573718	0.000152	1.89	V
*ERCC6L*	1.102811499	8.88E-05	0.79	N
*FANCA*	1.224811173	1.63E-06	4.33	V
*FOXM1*	1.92032584	2.17E-07	8.43	V
*HIST1H2AH*	1.485622497	0.000196	0.99	N
*HIST1H2AJ*	2.284502132	8.84E-06	2.96	V
*HIST1H2AM*	1.530373984	0.000151	1.98	V
*HIST1H2BH*	1.563333889	3.04E-07	1.32	V
*HIST1H3B*	2.979880506	6.94E-16	1.43	V
*KIF20A*	1.876073752	5.20E-08	8.09	V
*KIF23*	1.604227334	1.98E-09	3.22	V
*NCAPG*	1.888216879	2.37E-10	7.2	V
*PCNA*	0.939041116	1.44E-05	2.09	V
*RAD51*	1.255859843	1.79E-05	4.75	V
*RRM2*	2.285244825	5.90E-09	1.3	V
*TK1*	1.410412144	2.83E-05	5.67	V
*TPX2*	2.025558609	1.21E-10	5.49	V
*TYMS*	1.667388087	2.36E-09	5.64	V

TaqMan qPCR was performed for 29 genes representing the DNA replication and mitosis clusters. Twenty-seven of 29 were validated. RNA-Seq, whole transcriptome sequencing; V, validated; N, not validated.

### Ki-67 immunohistochemistry

Ki-67 immunohistochemistry shows strong (4) staining in the tissue sections from donors in the luteal phase or those using HC (Table S10 in Additional file 2). All sections demonstrating weak staining were from donors in the follicular phase. Intermediate staining (2 to 3) was mixed among both phases and donors using HC.

## Discussion

The functioning ovaries produce relatively large amounts of estradiol and progesterone in a cyclical pattern approximately every 28 days. The mid-cycle estrogen peak concentration can be 10 times that in the early follicular phase and progesterone serum concentrations are 10-fold higher in the luteal phase than follicular phase. In this study, next-generation RNA sequencing has been utilized to determine how these changes in serum steroid hormone concentrations effect breast epithelial gene expression. Gene expression in the breast epithelium of women using HC has also been assayed to determine the effects of these exogenous hormones. A total of 255 genes were differentially expressed when comparing the two phases of the menstrual cycle, of these 221 (87%) were more highly expressed during the luteal phase.

The specific changes in gene expression observed in the human breast epithelium as a function of the menstrual phase confirm and expand upon the results of Graham *et al.* who used both breast organoids and cell lines to test the effects of progesterone treatment [51]. The specific functions identified by those investigators were DNA replication, the G2/M checkpoint and kinetochore function, and the G1/S transition. A large percentage of the genes expressed during the luteal phase are cell cycle regulated and they can be broadly placed into one of two clusters: DNA replication and mitosis. The DNA replication cluster includes genes involved in DNA synthesis, including components of the pre-RC, nucleotide biosynthesis, DNA replication, DNA packaging, S phase regulation and DNA repair. The G_1_/S phase transcription phase regulator E2F1 binds to the promoter and thereby initiates the transcription of most genes in this cluster [32]. E2F1 transcription, in turn, is stimulated by the increased progesterone concentration during the luteal phase. This may be the direct result of the binding of the *E2F1* promoter by the progesterone receptor (PR) [52]. Progesterone may also act by increasing the expression of MYC [53], which subsequently facilitates, directly and indirectly, the expression of E2F1 [54,55]. The mitosis cluster contains genes involved in the processes of chromosome segregation, spindle organization, protein-DNA complex assembly, regulation of mitosis and cytokinesis. The promoters of most of these genes are bound by FOXM1. FOXM1 has a pivotal role in the regulation of cell proliferation and cell cycle progression. It exerts control on the G1/S transition, S-phase progression, DNA replication, centriole duplication, sister chromatid cohesion, G2/M transition, mitosis, DNA damage response and DNA repair [56]. Increased *FOXM*1 expression during the luteal phase is likely an indirect effect of the change in the concentration of ovarian steroids. Grant *et al.* state that E2F1 does not bind to the promoter of *FOXM1*[32], however, chromatin immunoprecipitation reveals binding of E2F1 to the proximal promoter of *FOXM1* in MCF-7 cells [57]. FOXM1 has been shown also to be regulated by TNFSF11 (RANKL) [58] and 14-3-3ζ [59]. Additional data reported by Bergamaschi, Katzenellenbogen and colleagues is also of interest: just over one-third of the 29 genes they identified to be significantly associated with 14-3-3ζ overexpression, tamoxifen resistance, FOXM1 expression, and the luminal B and a minority of basal subtypes of breast cancer have higher expression in the luteal phase [59]. The level of FOXM1 expression has also been shown to be correlated with the effectiveness of a number of other breast cancer therapies including herceptin [60], gefitinib [61], lapatinib [62], paclitaxel [60], and cis-platinum [63]. The downregulation of NUPR1, although not observed at the transcript level, is inferred by IPA upstream analysis. NUPR1, also known as p8 and COM1, is a chromatin-binding protein, which inhibits cell proliferation [64]. Reducing the expression of this protein accelerates the kinetics of G_1_ progression to S phase [64]. Clinical breast cancer studies have demonstrated significantly decreased p8 nuclear staining in breast cancer cells [65].

Only a small percentage of normal mammary cells are estrogen receptor (ER) and/or PR+ and mammary stem cells lack these receptors. Nevertheless, progesterone elicits significant changes in gene expression, which must be mediated by other factors. Brisken and colleagues have demonstrated that progesterone’s effect on mammary gland morphogenesis is mediated by WNT4 [66,67] and proliferation by RANK ligand [68], both acting by a paracrine mechanism. RANK ligand and WNT4 have been identified as paracrine effectors of progesterone-induced mammary stem cell expansion [18,19]. The data presented in this paper reveals increased expression of these genes during the luteal phase, likely a consequence of the increased progesterone concentration during this phase. Amphiregulin (AREG) has also been shown to be a downstream target of progesterone in the mouse mammary epithelium and its actions thought to be paracrine [69]. The expression of *AREG* was not increased during the luteal phase, however, another member of the epidermal growth factor family, epiregulin (*EREG*), was increased. EREG is an autocrine growth factor for normal human keratinocytes [70]. It can also induce the resumption of meiosis [71].

Asselin-Labat and colleagues utilized fluorescent-activated cell sorting to divide mouse mammary cells into luminal (CD29^lo^CD24^+^) and mammary stem cell-enriched (CD29^hi^CD24^+^) subfractions [18]. They then compared gene expression in these two subfractions as a function of ovariectomy. It is interesting to note that 54 (21%) of the genes differentially expressed as a function of the menstrual cycle are decreased as a consequence of ovarian hormone deprivation (Table S11 in Additional file 2). The reprise of these genes in the Asslin-Labat study is confirmatory evidence that the changes in the expression of these genes are indeed a consequence of the elaboration of ovarian hormones.

ESSRB expression was increased during the luteal phase. In *Drosophila* the activated single estrogen-related receptor ortholog causes a metabolic switch to a form of aerobic glycolysis that is reminiscent of the Warburg effect. It is tempting to speculate that a metabolic switch, such as occurs in *Drosophila,* provides the building blocks for the synthesis of amino acids, lipid and nucleotides required for the proliferation taking place during the luteal phase.

Many of the cell cycle genes identified with increased expression during the luteal phase have been shown to be overexpressed in breast cancer. This begs the question as to whether they have a role in oncogenesis or if the increased expression is simply a reflection of proliferating tumor cells. An increased number of ovulatory menstrual cycles and the prolonged use of progestin-containing hormone replacement therapy are both associated with an increased risk of the development of breast cancer [72,73]. Pike and colleagues hypothesized almost two decades ago that many of the epidemiologic observations regarding the relation of ovarian/steroidal hormones to breast cancer risk could be explained by an increased mitotic rate [14]. An increased mitotic rate may be increased mitotic activity above some baseline rate or it may be the division of a subset of cells that would ordinarily not be dividing, that is, stem cells. Joshi and colleagues clearly demonstrated that progesterone is driving the proliferation of murine mammary breast stem cells [19], and stem cells are likely to have accumulated a wealth of DNA lesions during their quiescence. Pike’s increased mitotic rate is likely a surrogate for cells re-entering and traversing the cell cycle. There are numerous redundant systems functioning during the cell cycle to ensure the correct replication of DNA and segregation of chromosomes; and, in those instances where repair cannot be affected, to eliminate those cells. Nevertheless, some of the products of the genes identified to have increased expression in the luteal phase genes have tight tolerances meaning there is a small margin of error. For example, both depletion and overexpression of KIAA0101 result in supernumerary centrosomes. The cell cycle is regulated by transcription, phosphorylation and ubiquitination events that must occur at a precise time within the cell cycle. Loss of this temporal control, such as the inappropriate expression of the progesterone-driven genes identified in the luteal phase, can result in genomic instability [74].

Oral contraceptives have been shown to leave the mitotic activity in the breast effectively unchanged when compared to a normal menstrual cycle and therefore have no chemopreventative effect [75]. These epidemiologic observations are substantiated by the findings of this sequencing study: there was only a single gene significantly differentially expressed when comparing the breast epithelium of women taking HC to women in the luteal phase of the menstrual cycle. The breast epithelium of a woman taking HC experiences a continuous luteal phase during the weeks that the breasts are exposed to the exogenous hormones. These hormones drive mitosis, cell cycle progression and proliferation using the same genetic programs as with endogenous ovarian hormones.

## Conclusions

Fundamental insights into the prevention of breast cancer are unlikely until we develop an understanding of the genetics and developmental biology of the normal mammary gland. The breast is one of the most complex genetic organs within the body. This is because DNA expression is under the control and influence of the hormonal milieu present in the circulation, which changes as a function of age; and for premenopausal women as a function of the menstrual cycle. We have taken advantage of a unique research resource: The Susan G. Komen for the Cure Tissue Bank at the IU Simon Cancer Center (KTB), a biorepository of normal, healthy breast tissue, to complete the first ever next-generation transcriptome sequencing of epithelial compartment of 20 normal human breast specimens. This work has produced an initial catalog of the differences in the expression of protein-coding genes as a function of the phase of the menstrual cycle. Gene expression in the breast epithelium was also compared between women using HC and those not. Gene expression increased in the luteal phase for the majority of the differentially expressed genes. The products of these genes regulate the cell cycle, mitosis, DNA licensing and replication, and the response to DNA damage including checkpoints and repair. Their expression is likely to have been a consequence of paracrine effectors of progesterone including RANKL, WNT4 and EREG. The breast epithelium of women using HC from the perspective of gene expression is that of a premenopausal woman during the luteal phase.

## Abbreviations

BER: base excision repair; bp: base pair; CCAN: constitutive centromere-associated network; CORE: Continuing Outcomes Relevant to Evista; CPC: chromosome passenger complex; DAB: 3,3′-diaminobenzidine; DAVID: database for annotation, visualization and integrated discovery; DE: differential expression; DNA: deoxyribonucleic acid; F: follicular; ER: estrogen receptor; FDR: false discovery rate; FFPE: formalin-fixed, paraffin-embedded; GO: Gene Ontology; GEF: guanine-nucleotide exchange factor; HC: hormonal contraception; IBIS-1: International Breast Cancer Intervention Study-1; IPA: Ingenuity Pathway Analysis; IU: Indiana University; kMT: kinetochore-microtubules; KTB: The Susan G. Komen for the Cure Tissue Bank at the IU Simon Cancer Center; L: luteal; LCM: laser capture microdissection; LH: luteinizing hormone; MCM: minichromosome maintenance; MORE: Multiple Outcomes of Raloxifene Evaluation; mRNA: messenger RNA; MT: microtubule; NB: negative binomial; NCI: National Cancer Institute; NSABP-P1: The National Surgical Adjuvant Breast and Bowel Project, Breast Cancer Prevention Trial 1; ORC: origin recognition complex; pre-RC: pre-replicative complexes; PR: progesterone receptor; RNA: ribonucleic acid; RNA-Seq: whole transcriptome sequencing; SAC: spindle assembly checkpoint; siRNA: small interfering RNA; STAR: Study of Tamoxifen and Raloxifene (STAR) Clinical Trial; TF: transcription factor.

## Competing interests

The authors declare that they have no competing interests.

## Authors’ contributions

SEC conceived and designed the study, analyzed the data and wrote the manuscript. SEC, TM, and AVS recruited normal volunteers and directed and maintained the Susan G. Komen for the Cure Tissue Bank at the IU Simon Cancer Center. IP, HAL, RJB, MRC, CAMS, DKD, TM, and BAH performed the laser capture microdissection and RNA extractions. DB assisted in data analysis. JG was responsible the experimental design of the next-generation sequencing; MH, JZ, and JG performed the next-generation sequencing. FZ, RWD, and YL performed the statistical analysis. MR participated in the microdissection of the breast tissue, study design and data analysis. RA participated in data analysis. SB reviewed histologic tissue sections and scored the immunohistochemistry. FZ, RWD, and SB drafted parts of the work; all authors participated in the revision of the various versions of the manuscript. All authors read and approved the final draft of this manuscript.

## Supplementary Material

Additional file 1 Experimental details of samples used, next-generation whole transcriptome sequencing, data analysis and qPCR validation. Click here for file

Additional file 2: Table S1 Description of data: experimental details, serum estriol concentrations, additional demographics of specimen donors. **Table S2.** Description of data: total number of sequencing reads and the reads able to be mapped to the human genome (hg 19). **Table S3.** Description of data: raw read counts of the individual genes. **Table S4.** Description of the data: differential gene expression luteal versus follicular. **Table S5.** Description of the data: functional annotation chart. **Table S6.** Description of the data: list of transcription factors identified by IPA Upstream Analysis. **Table S7.** Description of the data: luteal phase genes previously identified as FOXM1 target genes. **Table S8.** Description of the data: functions of a subset (34/47) of the genes involved in mitosis or cytokinesis [76-101]. **Table S9.** Description of the data: differential gene expression luteal versus hormonal contraception. **Table S10.** Description of the data: Ki-67 immunohistochemistry. **Table S11.** Description of the data: comparison of RNA-Seq data with that of Asselin-Labat and colleagues. List of genes identified to have differential expression as a function of ovarian hormones by both this study and that of Asselin-Labat *et al.*[18]. Click here for file
