# Supplementary material for: Next-generation transcriptome sequencing of the premenopausal breast epithelium using specimens from a normal human breast tissue bank
Source: Breast Cancer Res. 2014 Mar 17;16(2):R26. doi: 10.1186/bcr3627 (PMC4053088; doi:10.1186/bcr3627)
Supplement: Additional file 1 — Experimental details of samples used, next-generation whole transcriptome sequencing, data analysis and qPCR validation. [file bcr3627-S1.docx]

**SUPPLEMENTAL METHODS**

**Samples used for sequencing**

Fresh-frozen normal breast tissues (10 gauge cores) from healthy pre-menopausal volunteers with no history of breast cancer were procured from the Susan G. Komen for the Cure® Tissue Bank at the IU Simon Cancer Center. (Table 1 and Supplemental Table 1) Ductal epithelium was laser capture microdissected using various technologies. (Supplemental Table 1) Cores were embedded in OCT, sectioned (8µM thick) and the sections placed on PEN Membrane Glass Slides (Molecular Devices, Sunnyvale, CA). Cores were sectioned in their entirety and all sections were microdissected. Slides were kept at -80°C prior to dissection. Three slides were removed from the freezer at a time, and were stained using the HistoGene LCM Frozen Section Staining Kit (Arcturus, Life Technologies, Carlsbad, CA, USA). All dissections were completed within one hour of thawing to minimize RNA degradation. RNA from captured cells was extracted using the PicoPure RNA Isolation Kit (Arcturus) and quantified using the Qubit Fluorometer (Invitrogen, Carlsbad, CA, USA). Because of the lower yields associated with LCM, the entire amount of RNA was used for the RNA-seq library preparation.

**Next-Generation Whole Transcriptome Sequencing**

RNA samples were first enriched for the transcriptome by depleting the samples of large and small ribosomal RNA (rRNA) using the RiboMinus Eukaryote Kit (Invitrogen). Library preparation was then performed using the SOLiD Whole Transcriptome Analysis Kit or Total RNA-seq Kit (Life Technologies, Foster City, CA, USA). Each library was barcoded by using PCR primers containing different barcodes to allow multiple samples to be sequenced simultaneously on the Life Technologies SOLiD system. Emulsion PCR and bead preparation was conducted according to manufacturer’s instructions. Amplified beads were sequenced on the Life Technologies SOLiD system using 50bp fragment, or 50 x 35bp paired-end runs. Output data (.csfasta and .qual) files were converted to standard XSQ format for data analysis.

**Data Analysis**

*Read Mapping*

XSQ files containing the read sequences and quality values were loaded onto a compute cluster and the reads were mapped in colorspace using the Life Technologies LifeScope 2.5.1 software using default parameters. Reads were mapped to the human genome (hg19) downloaded from the UCSC Genome Bioinformatics Site (<http://genome.ucsc.edu>). The hg19 genome was slightly modified by deleting the Y chromosome in order to make a female genome. An hg19 exon reference file provided by Life Technologies was required by LifeScope in order to create the exon junction libraries needed to map reads that cross exon boundaries. This file was derived from the refGene database from UCSC. This file also served to provide the gene model needed to derive count data for differential expression. Also, a human filter reference file was required (provided by Life Technologies) that contains the sequences of ribosomal and repetitive regions of the genome in order to filter reads that mapped to those regions. Mapped reads were outputted in the standard BAM (Binary Alignment/Map) format. Count data on a gene-by-gene basis was provided through a text file produced by LifeScope.

Statistical Analysis

*Negative Binomial Model*

Differential expression (DE) was tested using the Bioconductor package *edgeR* in R (v. 2.15). A negative binomial (NB) distribution was employed to model the count data generated from the RNA-Seq experiments. Note that use of this distribution acknowledges the over-dispersion issue present in next-gen count data (i.e., the mean and the variance are not equal). The data are distributed as *Y_gijk_ ~ NB(M_k_ p_gij_, φ_g_),* where *Y_gijk_* is the number of reads from group *i* (contraceptive, luteal, or follicular) and batch *j* that are mapped to gene *g*; *M_k_* is the total number of mapped reads for sample *k*; *p_gij_* is the proportion of all reads that originate from gene *g* in the *i^th^* group and *j^th^* batch; and *φ_g_* is the dispersion parameter for gene *g*.

A set of three hypotheses were tested using three similar general linear models. Model (1) is used to determine differential expression between women using hormonal contraceptives and women in the luteal phase of the menstrual cycle. The general linear model for this analysis is denoted

$\log(Y_{gk})=\beta_{1g}L_{k}+\beta_{2g}Batch2_{k}+\beta_{3g}Batch3_{k}+\log M_{k}+\varepsilon_{gk}$ (1)

where *Y_gk_* is the observed counts for gene *g* in sample *k; L_k_* is an indicator variable for membership of sample *k* in the luteal group, with membership in the contraceptive group being used as the baseline for comparison; Batch2*_k_* and Batch3*_k_* are indicator variables for memberships of sample *k* in batches 2 and 3, with membership in batch 1 being used as the baseline for comparison; log*M_k_* is an offset term representing the sample *k* library size; and $\varepsilon_{gk}$ is the error term.

Model (2) is used to determine differential expression between women using hormonal contraceptives and women in the follicular phase of the menstrual cycle. The primary purpose of this model is to see whether there will be more differentially expressed genes between this comparison and the comparison in Model (1). The general linear model for this analysis is denoted

$\log(Y_{gk})=\beta_{1g}F_{k}+\beta_{2g}Batch2_{k}+\beta_{3g}Batch3_{k}+\log M_{k}+\varepsilon_{gk}$ (2)

where *F_k_* is an indicator variable for membership of sample *k* in the follicular group, with membership in the contraceptive group being used as the baseline for comparison; and $\varepsilon_{gk}$ is the error term.

Model (3) is used to determine differential expression between women in the two different phases of the menstrual cycle: luteal and follicular. The general linear model for this analysis is denoted

$\log(Y_{gk})=\beta_{1g}F_{k}+\beta_{2g}Batch2_{k}+\beta_{3g}Batch3_{k}+\log M_{k}+\varepsilon_{gk}$ (3)

where *F_k_* is an indicator variable for membership of sample *k* in the follicular group, with membership in the luteal group now being used as the baseline for comparison; and $\varepsilon_{gk}$ is the error term.

For each of these three models, we are testing for each gene *g* the null hypothesis that there is no effect of membership in one group in comparison to the baseline group. The hypotheses are denoted as follows: For gene g, $H_{0}:\beta_{1g}=0$;$H_{a}:\beta_{1g}\neq0$

The test statistic is equivalent to

*F_g_ = LRT_g_ φ_g_ ~F_1,n-p_* under H_0_  (4)

where  *LRT_g_* is the quasi likelihood test statistic; *φ_g_*is the dispersion parameter estimation in the NB model; *n* is the sample size; and *p* is the number of parameters estimated in the model. From (4) we can see that accurately estimating the dispersion parameter is important since underestimating *φ_g_*tends to cause lower p-values, resulting in a higher false discovery rate.

The NB distribution works well for RNA-Seq data, since it is a discrete distribution and thus appropriate for count data. While a Poisson distribution could also model counts, the variance is specifically assumed to be equal to the mean, which is generally not true for RNA-Seq data. We are allowed more flexibility in NB model due to the additional use of the dispersion parameter, *φ_g_*. This parameter allows for a more accurate modeling of the variability between samples. Note that when the *φ_g_* is zero, the NB model reduces to the Poisson model.

**Validation**

*qPCR*

TaqMan qPCR was performed using the following inventoried TaqMan assays from Applied Biosystems (Life Technologies):

| **Gene Symbol** | **Assay ID** | **Gene Name** |  |
| --- | --- | --- | --- |
| 18S | Hs99999901_s1 | Eukaryotic 18S rRNA |  |
| AURKB | Hs00177782_m1 | aurora kinase B |  |
| BRCA1 | Hs01556193_m1 | breast cancer 1, early onset |  |
| BUB1B | Hs01084828_m1 | budding uninhibited by benzimidazoles 1  homolog beta (yeast) |  |
| BUB1 | Hs00177821_m1 | budding uninhibited by benzimidazoles 1  homolog (yeast) |  |
| CCNB1 | Hs01030097_m1 | cyclin B1 |  |
| CDC25C | Hs00156411_m1 | cell division cycle 25 homolog C (S. pombe) |  |
| CDC6 | Hs00154374_m1 | cell division cycle 6 homolog (S. cerevisiae) |  |
| CDCA8 | Hs00983655_m1 | cell division cycle associated 8 |  |
| CDK1 | Hs00938777_m1 | cyclin-dependent kinase 1 |  |
| CENPE | Hs00156507_m1 | centromere protein E, 312kDa |  |
| CLSPN | Hs00898637_m1 | claspin homolog (Xenopus laevis) |  |
| E2F1 | Hs00153451_m1 | E2F transcription factor 1 |  |
| ECT2 | Hs00216455_m1 | epithelial cell transforming sequence 2 oncogene |  |
| ERCC6L | Hs00535177_s1 | excision repair cross-complementing rodent repair  deficiency, complementation group 6-like |  |
| FANCA | Hs01116668_m1 | Fanconi anemia, complementation group A |  |
| FOXM1 | Hs01073586_m1 | forkhead box M1 |  |
| GAPDH | Hs99999905_m1 | glyceraldehyde-3-phosphate dehydrogenase |  |
| HIST1H2AH | Hs00544732_s1 | histone cluster 1, H2ah |  |
| HIST1H2AJ | Hs00544489_s1 | histone cluster 1, H2aj |  |
| HIST1H2AM | Hs00361889_s1 | histone cluster 1, H2am |  |
| HIST1H2BH | Hs00374322_s1 | histone cluster 1, H2bh |  |
| HIST1H3B | Hs00605810_s1 | histone cluster 1, H3b |  |
| HPRT1 | Hs99999909_m1 | hypoxanthine phosphoribosyltransferase 1 |  |
| KIF20A | Hs00993573_m1 | kinesin family member 20A |  |
| KIF23 | Hs00370852_m1 | kinesin family member 23 |  |
| NCAPG | Hs00254617_m1 | non-SMC condensin I complex, subunit G |  |
| PCNA | Hs00427214_g1 | proliferating cell nuclear antigen |  |
| PPIA | Hs99999904_m1 | peptidylprolyl isomerase A (cyclophilin A) |  |
| RAD51 | Hs00153418_m1 | RAD51 homolog (RecA homolog, E. coli) (S. cerevisiae) |  |
| RRM2 | Hs01072069_g1 | ribonucleotide reductase M2 |  |
| TK1 | Hs01062125_m1 | thymidine kinase 1, soluble |  |
| TLR6 | Hs00271977_s1 | toll-like receptor 6 |  |
| TOX2 | Hs00262775_m1 | TOX high mobility group box family member 2 |  |
| TP73 | Hs01056230_m1 | tumor protein p73 |  |
| TPX2 | Hs00201616_m1 | TPX2, microtubule-associated, homolog (Xenopus laevis) |  |
| TYMS | Hs00426586_m1 | thymidylate synthetase |  |

Briefly, 20ng of RNA from each sample of the validation cohort was reversed transcribed using the High Capacity RNA-to-cDNA kit (Applied Biosystems). cDNA was then pre-amplified using TaqMan PreAmp Master Mix (Applied Biosystems) and the TaqMan assays per manufacturer’s instructions. qPCR of target genes and housekeepers was performed in triplicate using TaqMan low density arrays (Applied Biosystems) spotted with the above listed assays. qPCR reactions were run on an ABI 7900HT Real-Time PCR System (Applied Biosystems) and data analyzed using the SDS2.3 and DataAssist v2.0 software from Applied Biosystems. Fold change was calculated using the standard ΔΔCt method incorporating the geometric mean of the housekeepers (18S, GAPDH, HPRT, and PPIA).
